# Intestinal Dendritic Cells in Health and Gut Inflammation

**DOI:** 10.3389/fimmu.2018.02883

**Published:** 2018-12-06

**Authors:** Andrew J. Stagg

**Affiliations:** Centre for Immunobiology, Blizard Institute, Barts and The London School of Medicine and Dentistry, Queen Mary University of London, London, United Kingdom

**Keywords:** Dendritic cells, intestinal inflammation, antigen sampling, lymphocyte homing, inflammatory bowel disease

## Abstract

Dendritic cells (DCs) mediate tolerance to food antigens, limit reactivity to the gut microbiota and are required for optimal response to intestinal pathogens. Intestinal DCs are heterogeneous but collectively generate both regulatory and effector T cell responses. The balance of outcomes is determined by the activity of functionally distinct DC subsets and their modulation by environmental cues. DCs constantly sample luminal content to monitor for pathogens; the significance of the various pathways by which this occurs is incompletely understood. Intestinal DC have distinctive properties shaped by local host, dietary and microbial signals. These properties include the ability to produce all-trans retinoic acid (RA) and imprint gut tropism on T cells they activate. In the steady-state, subsets of intestinal DC are potent generators of inducible Treg, aided by their ability to activate TGFβ and produce RA. However, responses induced by steady-state intestinal DCs are not exclusively regulatory in nature; effector T cells with specificity for commensal bacterial can be found in the healthy mucosa and these can be locally controlled to prevent inflammation. The ability of intestinal DCs to enhance effector responses in infection or sustain inflammation in disease is likely to involve both modulation of the local DC population and recruitment of additional populations. Immune pathways in the pathogenesis of inflammatory bowel disease can be mapped to DCs and in inflamed intestinal tissue, DCs show increased expression of microbial recognition machinery, activation, and production of key immunological mediators. Intestinal DCs may be targeted for disease therapy or to improve vaccine responses.

## Introduction

Dendritic cells (DCs) are bone marrow-derived antigen presenting cells which comprise two major subsets: conventional (or classical) DCs (cDCs) and plasmacytoid DC (pDC). They are developmentally distinct from both tissue resident macrophages and monocyte-derived populations (1) but share many phenotypic markers with these populations. Historically this has led to confusion and apparently conflicting data in analyses of DCs in the intestine. However, recent development of better strategies for DC identification, together with improved cell isolation techniques that help maintain DCs in their native state (2, 3), and genetic tools that enable specific deletion of DC populations *in vivo*, have enabled a clearer picture of the role of DCs in the intestine to emerge.

cDCs play critical roles in immune regulation in the intestine and are the focus of this review. The reader is referred to a recent review article (4) for more information on the role of pDC in the intestine. cDCs are required for induction of oral tolerance (5) and the generation of regulatory T cells (Treg) recognizing soluble antigens (5) and commensal microbes (6, 7). They are also required for optimal protective immune responses against diverse pathogens (8–12). The balance of regulatory and effector responses is influenced by the contribution of functionally distinct cDC subsets as well as their modulation by environmental cues.

## Intestinal Dendritic Cells in the Steady-State

### Heterogeneity of Intestinal DCs

Intestinal cDCs are found within organized lymphoid tissue, including Peyer's patches (PP) (13) and draining lymph nodes, as well as in the lamina propria (LP) of small intestine (SI) and colon (14–16). As in other tissues, LP cDCs comprise cDC1 and cDC2 which can be defined in mice and humans by expression of X-C motif chemokine receptor 1 (XCR1) or signal regulatory protein α (SIRPα/CD172a), respectively, (1, 17).

**Figure 1:**
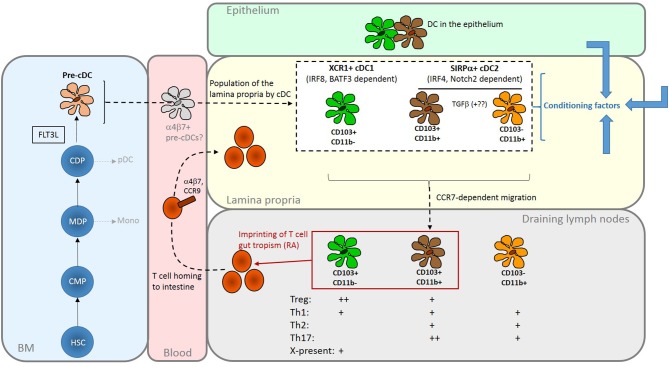
Summary of the origins and properties of intestinal DC. FLT3L-dependent development of cDC gives rise to two major subsets via CDP and committed pre-cDC in the BM. cDC1 are XCR1+ and depend on transcription factors including IRF8 and BATF3; cDC2 are SIRPα+ and depend on transcription factors that include IRF4. Population of the intestinal mucosa may be via an intermediate gut-committed α4β7+ precursor [sometimes termed pre-mucosal (μ) DC] stage (19, 20). In the mouse LP, the cDC2 population comprises two closely related CD11b+ subsets that can be distinguished by the presence or absence of CD103; CD11b+CD103+, found predominately in the SI, are probably derived from the CD11b+CD103– cells under the influence of TGFβ. The cDC1 population is CD11b–CD103+. Similar populations in the human intestine can be identified on the basis of CD103 and SIRPα expression. In the LP they are exposed to conditioning factors (shown as blue arrows) from epithelial cells and other sources which further shape their functional properties. All three subsets have the capacity to migrate to draining lymph nodes to interact with recirculating T cells. Here, production of RA by LP- derived DCs, particularly those expressing CD103, imprints gut tropism on T cells they activate by inducing expression of α4β7 integrin and the chemokine receptor CCR9. Although DCs can induce both Treg and effector T cells, with some specialism across DC subsets, the balance of these outcomes favors regulation in the steady-state. The reader is referred to recent reviews for a more in-depth discussion of intestinal DC subsets (21, 22). HSC, haematopoietic stem cell; CMP, common myeloid progenitor; MDP, monocyte dendritic cell precursor; CDP, common dendritic cell precursor; pDC, plasmacytoid DC; Mono, monocyte; X-present, cross-presentation to CD8+ T cells.

Additional cell surface markers are used in particular species to define intestinal cDC subsets further. In mice, cDC1 are CD103+CD11b–, whereas cDC2 comprise CD103–CD11b+ cells and a gut-specific CD103+CD11b+ population (18) (Figure 1). The two CD11b+ populations are closely related; CD103–CD11b+ cDC give rise to CD103+CD11b+cells under the influence of TGFβ (23). There are more CD103+CD11b+ in the SI than in the colon (24). Equivalent cDC populations are present in the human intestine (25) and are often identified based on expression of CD103 in conjunction with SIRPα rather than CD11b.

LP DC populations are dependent on FLT3L for development and are derived from a committed pre-cDC progenitor (Figure 1). Some pre-cDC can acquire expression of α4β7 integrin and thereby commit to an intestinal cDC fate (16, 19, 20). LP cDCs express the cDC-specific transcription factor Zbtb46 (11) but not the macrophage marker CD64. Critically, all have the capacity to migrate in a CCR7-dependent manner to draining LN to interact with recirculating T cells (26, 27). LP cDC subsets require different transcription factors for development and their selective deletion has enabled some of their functions to be defined (Figure 1). Detailed discussion of these experiments is beyond the scope of this review but the reader is referred to other recent authoritative articles (21, 22).

### Antigen Sampling

To protect against luminal pathogens and establish regulatory response to innocuous antigens, DCs continuously sample intestinal contents (28, 29). In PP, microfold (M) cells in the follicle associated epithelium internalize bacteria and other particulates and deliver them to underlying DCs [reviewed in (30)]. PP cDCs can also capture translocated IgA immune complexes (31) and extend dendrites through M cell specific transcellular pores (32). cDCs cross-present viral antigen captured from infected epithelial cells (33, 34). Ileal CD103+ cDCs in the epithelium (Figure 1) sample soluble and particulate antigen (35). Transport of low molecular weight soluble material by SI goblet cells (36) and retro-transport of IgG immune complexes across the epithelium (37) can also deliver antigen to cDCs. Non-migratory CX3CR1+ macrophages in the SI sample antigens via trans-epithelial processes (38–41) and hand on to migratory CD103+ cDCs (38). The functional significance of different modes of sampling and the impact of inflammation are poorly understood.

### Imprinting of Gut Tropism

Lymphocytes activated in gut lymphoid tissue traffic to the intestinal mucosa because they express specific adhesion molecules and chemokine receptors. The integrin α4β7 binds MAdCAM-1 expressed by intestinal vascular endothelium and facilitates entry to both the colon and the SI. The chemokine receptor CCR9 is required for entry to the SI, where its ligand CCL25 is expressed (42). GPR15 is implicated in homing to the colon but there may be important differences between mice and humans with regard to its role in the trafficking of effector vs. regulatory T cell populations (43–45). These mechanisms facilitate lymphocyte homing to the intestine in the absence of overt inflammation and enable intestinal responses to be regulated independently of the systemic response. In mice, cDCs that have migrated from LP to mesenteric lymph nodes induce expression of α4β7 and CCR9 on T cells they activate (46–49) via production of all-trans retinoic acid (RA) (50). Only cDC from the intestine express the enzyme RALDH2 (encoded by *aldh1a2*), required for the generation of RA from dietary vitamin A, explaining the site-specific induction of gut tropism (50). Initial data suggested that only CD103+ cDC had imprinting activity (15, 46, 51, 52) but it has subsequently been detected in mouse CD103– cDCs (26). Also, both CD103+ and CD103– cDCs in the human colon express *ALDH1A2* (53). Stromal cells in mesenteric lymph nodes can also produce RA to reinforce the imprinting activity of migratory intestinal cDCs (54–56).

### Induction of Regulatory and Effector T Cell Responses

In the steady-state, intestinal DCs can induce Treg. In the mouse SI, induction of gut tropic Treg directed against soluble antigens, by both CD103+CD11b+ and CD103+CD11b– DCs, occurs in the mesenteric LN (52) and underlies the long-recognized phenomenon of oral tolerance generated to such antigens (57). The ability of SI CD103+ cDC to generate Treg is dependent on their expression of the integrin αvβ8, which activates latent TGFβ, and is enhanced by their production of RA (58–62). PD-L1 and PD-L2 have also been implicated in generation of Treg by MLN cDC (63). It is notable that induction of tolerance to colonic antigens differs from tolerance in the SI in that it is induced in the iliac, not mesenteric, nodes, is mediated by CD103–CD11b+ cDC and is independent DC-generated RA (16). The generation of Treg directed against commensal bacteria has been less studied. Nonetheless, in a cell transfer model, the rapid generation of Treg from naïve commensal-reactive transgenic CD4 T cells required Notch2-dependent but not Batf3-dependent cDC, suggesting that SIRPα+ cDC2, possibly CD103+CD11b+ cells, play a dominant role (7).

T cell responses stimulated by DCs in the steady-state are not exclusively regulatory. Effector T cells are present in the lamina propria of healthy mice and humans; although some of these may reflect past pathogen encounter others are specific for the commensal microbiota (64, 65). Effector cells in the healthy intestine enhance the epithelial barrier (66) and protect against translocation of pathogens (67). Their activity can be locally controlled by regulatory CX3CR1^hi^ mucosal myeloid populations (68), anti-inflammatory cytokines such as TGFβ (69) as well as Treg. CD103– cDC migrating from the mouse SI can prime effector T cells in the absence of stimulation (26) indicating one mechanism by which these responses can be generated.

### Conditioning of Intestinal DC

The ability of intestinal cDC to generate RA and promote tolerance requires active Wnt/β-catenin signaling with the cDCs (70) and is determined in part by local environment cues (71). Epithelial cells promote the ability of DC to generate both regulatory (72, 73) and Type 2 responses (74). In the mouse, epithelial TSLP, with IL-25 and IL-33, inhibits IL-12 production by DCs and promotes their ability to generate Th2 responses that clear *Trichuris muris* infection (74). RA and TGFβ from human epithelial cells promote regulatory DC function (72). Exposure to RA can induce characteristics of SI DCs *in vitro* (75) and is required for *aldh1a2* expression (76). Sources of RA include epithelial cells (77), LP stromal cells (78), and bile retinoids (79). In contrast, prostaglandin E2 has been reported to negatively regulate the expression of RA generating enzymes in DC (80). Dietary and microbial products, including ligands of the aryl hydrocarbon receptor [AhR (81)] and butyrate (82), also affect intestinal DCs.

## Intestinal Dendritic Cells in the Promotion of Effector Function and Inflammation

### Promotion of Effector Function

The balance of responses induced by DC can change in the context of infection to favor effector mechanisms. Signaling through p38 MAPK in CD103+ mouse DC regulates the balance of Treg and Th1 development from naïve T cells (83); in its absence, expression of RALDH2 and generation of Treg are reduced but Th1 responses enhanced.

**Figure 2:**
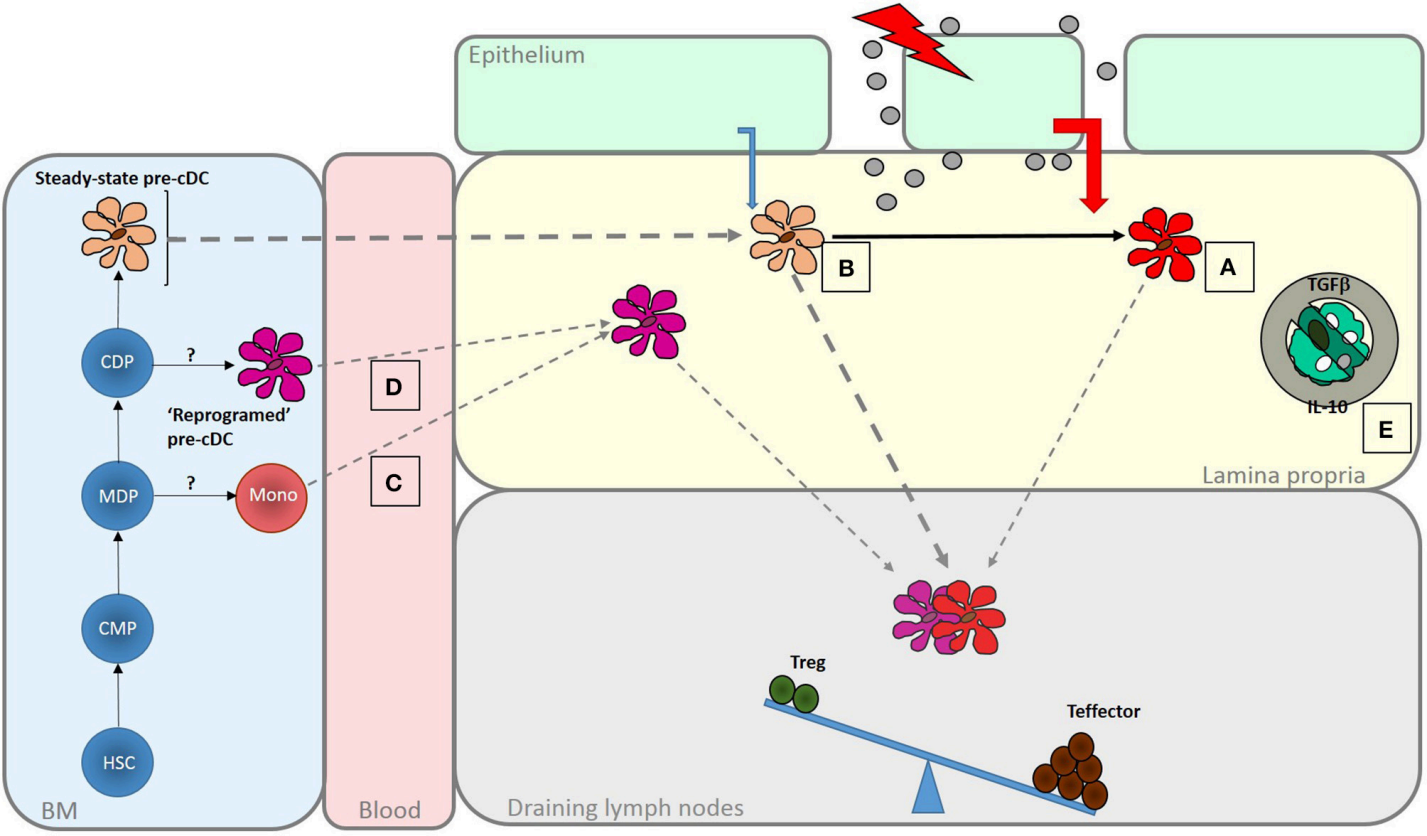
Enhanced generation of effector T cells in response in infection and inflammatory disease. A number of mechanisms act to shift the balance of response induced by intestinal DC to favor the generation of effector responses. These are important for pathogen clearance but if dysregulated may lead to inflammatory disease. Increased exposure to PAMPS and DAMPS, as the result of loss of barrier integrity or tissue penetration by pathogens may be sensed by LP DC populations leading to their activation **(A)**. Altered microbial sensing by epithelial cells due, for instance, to engagement of basolateral PRRs, may change the nature of conditioning factors they produce. Increased DC turnover may reduce exposure to those conditioning factors that normally promote regulatory function in intestinal DC **(B)**. Alternatively, different types of DC, specialized to promote effector responses, may be recruited to the intestine under these conditions. These could be DC-like cells derived from monocytes **(C)** or possibly “reprogrammed” DC arise as a result of altered differentiation in the BM **(D)**. There may also be a release from the regulatory mechanisms that normally limit effector function within the mucosa, due, for instance to a loss of response to regulatory cytokines like IL-10 or TGFβ or a failure to interact with CX3CR1+ cells that promote regulation **(E)**. The role of DCs on determining responsiveness to these local control mechanisms is not known.

A change in the balance of T cell responses induced by intestinal cDC may result from direct modulation by danger signals, altered conditioning of the resident cDC population or recruitment of distinct pro-inflammatory DC (Figure 2). Intestinal cDC express pattern recognition receptors and respond to microbial products (84, 85). Subsets of mouse and human DC differentially express Toll-like receptors (TLRs) suggesting specialization for direct recognition of particular microbes (25, 86). Mouse CD103+CD11b+ cDCs express TLR5 and their ability to induce Th17 responses is enhanced following activation with flagellin (24, 85, 87, 88). Activation of CD103+CD11b+ cDC results in increased production of IL-6 and IL-23 which promote Th17 development and production of the anti-microbial peptide RegIIIγ (85, 89). Administration of a TLR7 agonist *in vivo* results in activation of a CD103+CD11b– cDC migratory subset with the ability to generate effector CD8+ T cell responses to cross-presented antigen (90).

In most tissues, exposure to microbial products is sufficient to convert immature cDCs to mature cells which generate potent effector responses. However, exposure to PAMPs from the commensal microbiota is likely to be a common occurrence in the healthy intestine and therefore a second signal may also be required. Indeed, mouse CD103+ SI cDCs can induce Treg even in the presence of the high level of costimulatory molecule expression characteristic of mature cDC (58). The nature of this second signal is not known but IgA-containing immune complexes, normally restricted to the lumen, but present in significant quantities in damaged tissues, can enhance the pro-inflammatory activity of DCs (91).

Intestinal cDCs reside in the mucosa for a few days (92) during which time they are conditioned to acquire regulatory properties. cDCs that escape conditioning in the steady-state may facilitate the generation of “tonic” protective effector T cell responses. An increase in turnover following exposure to TLR ligands (93, 94) or inflammatory cytokines could shorten residence time, reduce exposure to conditioning factors and increase cDC-generated effector responses.

Intestinal cDC function may also be influenced remotely during their development in the bone marrow. Intestinal inflammation alters hematopoiesis to influence the development of monocytes and subsequently the intestinal populations derived from them (95). These concepts remain to be explored for cDCs but changes in blood DCs have been described in IBD (96, 97).

Alternative precursors may also be recruited into the tissue under inflammatory conditions to provide cells with the ability to generate effector responses. Monocytes can give rise to DC-like cells (monocyte-derived DC; moDC) under inflammatory conditions (98, 99). In healthy mice, monocytes recruited into the intestine differentiate into anti-inflammatory macrophages (100–103). However, under inflammatory conditions this differentiation process is interrupted, generating a population with some DC-like properties including the ability to activate naïve T cells and generate Th1 cells (102). Similarly, cells expressing the monocyte marker CD14 accumulate in the inflamed mucosa of inflammatory bowel disease (IBD) patients (104, 105) and these too have a high capacity to naive stimulate T cells and generate gut tropic Th1 cells and Th17 cells (104). However, it is unclear if monocyte derived-cells can migrate to lymphnodes or act solely within the mucosa.

Effector cells generated by DCs could also be released from local control within the tissues where diverse antigen presenting cell (APC) populations exert a local influence (68, 106–110). In the context of chronic inflammation, T cells lose responsiveness to regulatory TGFβ due to over-expression of SMAD7, an inhibitor of TGFβR signaling (69). In addition, inflammatory cytokines can change the repertoire of peptides presented by DCs (111) and the emergence of “cryptic” determinants may allow escape from Treg control.

Irrespective of the combination of mechanisms which allow effector responses to be enhanced to deal with infections, a key unanswered question remains how these responses are confined to the pathogen and do not normally extend to co-sampled commensal organisms.

### DC and Inflammatory Intestinal Disease

Many immune pathways key to IBD pathogenesis, highlighted by the identification of genetic variants associated with disease susceptibility, can be mapped to DCs. Nucleotide-binding oligomerization domain–containing-2 (NOD2), loss of function variants of which are associated with Crohn's disease (CD), is a bacterial sensor in DCs; its engagement impacts upon bacterial handling, cytokine production and antigen presentation (112, 113). DCs that express CD-associated variants are defective in these pathways (112, 113). The IL-23 axis is implicated in CD and DCs are a major source of bacterially driven intestinal IL-23 (85, 87, 89). Moreover, expression of genes associated with variation in CD prognosis, rather than susceptibility, can also be mapped to DCs (114).

Mouse models of colitis provide direct evidence for the importance of DCs. Transfer of bone marrow derived DCs increases inflammation whereas depletion of DCs reduces it (115, 116). Colitis develops in mice in which TGFβ receptor signaling is non-functional in DCs (23, 117) and, in T cell deficient mice, administration of an agonistic anti-CD40 antibody activates DCs and induces IL-23-dependent intestinal inflammation (118).

DCs isolated from inflamed intestinal tissue show evidence of enhanced microbial recognition and heightened activation. More colonic cDCs from inflamed tissue of IBD patients express TLR2 and TLR4 (119, 120) and they also express higher levels of activation-associated CD40 (119). In CD, mature CCR7+ DCs, retained in the mucosa by locally produced ligands, cluster with proliferating T cells (121).

In CD, more colonic LP cDCs produce IL-12/23p40 and IL-6 but the proportion of cDCs that produce IL-10 is similar to healthy controls (119). Production of pro-inflammatory cytokines by colonic cDCs in CD correlates with disease activity, levels of inflammation and aspects of the intestinal microbiota (122). In UC, the frequency of cDCs producing IL-12/23p40 and IL-10 has been reported to be greater (122) or similar (119) to healthy controls in different studies. Production of IL-6 by cDC from UC patients was not increased in either study. In coeliac disease, activated DC producing Th1-promoting cytokines accumulate in the duodenal mucosa (123). In the MLN of CD patients, DCs release more IL-23 upon bacterial stimulation and CD4+ T cells produce more IL-17 and IFNγ (124).

MicroRNAs are short non-coding RNA molecules that function in RNA silencing and post-transcriptional regulation of gene expression. Expression of microRNA (miR)-10a, which acts in DC to limit their ability to produce IL-23/23p40 and generate Th1 and Th17 responses, is reduced in the inflamed mucosa of IBD patients (125). The IBD-associated reduction in mir-10a is accompanied by increased expression of IL-12/23. In UC, immature colonic DCs are poorly stimulatory but induce an atypical T cell response characterized by increased IL-4 but reduced IFNγ and IL-22 (120, 126) possibly indicating a reduced ability to induce barrier protective T cell responses.

Numbers of CD103+ cDCs are reduced in mice with chronic ileitis (127) and in the colon of UC patients (128). Whether these differences reflect a change in phenotype or altered cDC populations is not clear. Unlike equivalent cDCs from normal human colon, CD103+ colonic cDCs from UC patients do not generate Foxp3+ Treg but do generate IFNγ-, IL-13-, and IL-17-producing CD4 T cells. CD103+ cDCs from UC patients also have higher expression of IL-6, IL-12p40, IL-12p35, and TNFα (128).

RA is required for optimal Treg induction by intestinal CD103+ cDCs in the steady-state but these cells have reduced expression of RA-generating enzymes in mice with colitis (127, 129). The effects of RA on the immune system are context dependent [reviewed in (130, 131)] and it can promote pro-inflammatory as well as regulatory responses (132). In the presence of IL-15, RA induces the release of pro-inflammatory IL-12 and IL-23 by cDC and promotes intestinal inflammation (133). There is conflicting data on the production of RA by DC in the inflamed human colon: increased (53) and decreased (134) expression of RA-generating enzymes have both been reported in IBD. It should be borne in mind that neither study measured RA itself nor assessed other factors that regulate RA availability, such as expression of CYP26 enzymes that degrade RA (135).

## Concluding Remarks

Antibodies which target α4β7, an integrin which can be imprinted by intestinal DCs to facilitate T cell entry to the intestine, are already used to treat IBD. DCs also control the balance between regulatory and effector T cell responses in the intestine. Understanding the mechanisms involved and their regulation will facilitate rational manipulation of DCs to promote effector responses in the context of infection and vaccination or to re-establish regulation in the context of inflammatory disease.

## Author Contributions

The author confirms being the sole contributor of this work and has approved it for publication.

### Conflict of Interest Statement

The author declares that the research was conducted in the absence of any commercial or financial relationships that could be construed as a potential conflict of interest.
